# Role of MicroRNAs in Breast Cancer Metastasis to the Brain: A New Therapeutic Perspective

**DOI:** 10.31661/gmj.v12i.3193

**Published:** 2023-12-01

**Authors:** Baback Khanegheini, Afsaneh Ghasemi, Mohammad Amin Heidari, Kamkar Aeinfar, Sina Firoozi, Mona Tamaddon, Zhila Fereidouni

**Affiliations:** ^1^ Brain and Spinal Cord Injury Research Center, Neuroscience Institute, Tehran University of Medical Sciences, Tehran, Iran; ^2^ Department of Public Health, School of Health, Fasa University of Medical Sciences, Fasa, Iran; ^3^ Department of Pharmacology, Faculty of Medicine, Ilam University of Medical Science, Ilam, Iran; ^4^ Department of Neurosurgery, Shariati Hospital, Tehran University of Medical Sciences, Tehran, Iran; ^5^ School of Medicine, Kermanshah University of Medical Sciences, Kermanshah, Iran; ^6^ Chronic Disease Research Center, Endocrinology and Metabolism Population Sciences Institute, Tehran University of Medical Sciences, Tehran, Iran; ^7^ Department of Nursing, School of Nursing, Fasa University of Medical Sciences, Fasa, Iran

**Keywords:** Breast Cancer, Micro RNA, Prognosis, Treatments, Brain Metastasis

## Dear Editor,

MicroRNAs (miRNAs) are small non-coding RNA molecules that regulate gene expression by binding to the messenger RNA (mRNA) of specific genes [1]. In recent years, evidence has demonstrated the role of miRNAs in various aspects of cancer progression, including metastasis [2]. Indeed, their ability to influence multiple signaling pathways involved in tumor growth, angiogenesis, invasion, and immune response highlights their significance in cancer biology [3].Breast cancer (BC) metastasis to the brain poses a formidable clinical challenge, resulting in poor patient outcomes and limited treatment options [4]. Recent research has indicated that specific miRNAs are implicated in this process, either promoting or suppressing brain metastasis formation [5]. For example, miR-10b has been identified as a metastasis-promoting miRNA, influencing tumor invasiveness and enhancing colonization of BC cells to the brain by targeting various genes involved in cell adhesion and angiogenesis [6]. Additionally, miR-520h has been shown to suppress the expression of genes associated with the epithelial-mesenchymal transition, thus inhibiting BC cell migration and invasion to the brain [6]. Table-1 indicates some important miRNAs [7],[8],[9],[10],[11],[12],[13],[14] with the propensity of brain metastasis among patients with BC.Furthermore, Jordan-Alejandre et al. [15] revealed the potential of miRNAs as prognostic biomarkers in BC patients with brain metastasis (Table-2). For instance, elevated circulating levels of miR-210 have been correlated with an increased risk of brain metastasis, suggesting its utility as a predictive biomarker for identifying patients at higher risk of developing brain metastasis [16]. Hence, manipulating the expression levels of specific miRNAs and/or targeting miRNA-mRNA interactions could provide novel therapeutic strategies [17]. Although several pre-clinical studies [17, 18] have demonstrated encouraging results in inhibiting metastasis by either delivering synthetic miRNA mimics or using anti-miRNA agents to suppress oncogenic miRNAs (Table-3), further elucidation of the intricate interplay between miRNAs and their target genes is essential for the successful translation of these findings into clinical practice.Also, close collaboration and interdisciplinary efforts among neurosurgeons, researchers, oncologists, geneticists, and bioinformatics are necessary to integrate miRNA-based approaches into standard clinical practice.In conclusion, the role of miRNAs in BC metastasis to the brain represents a potential treatment approach. Hence, by investigating the complex networks of miRNA-based molecular alterations and their potential role as prognostic biomarkers and therapeutic targets, we could provide more effective personalized strategies to reduce metastasis rates, especially to the brain, in patients with BC.

**Table:** Table1. Some miRNA and Targeted Genes Involved in Brain Metastasis Among Patients with BC

**miRNAs**	**Targeted Genes**	**Functions**	**Ref**
miR-10b	*HOXD10, TP53*	Promotes invasion, metastasis, and angiogenesis	[7]
miR-210	*EFNA3, PTEN, RAD52*	Induces angiogenesis and enhances cell survival	[8]
miR-122	*ADAM10, PKM2*	Affects tumor growth, migration, and metabolism	[9]
miR-127	*BAI1, ZEB1, MMP16*	Suppresses metastasis and inhibits EMT	[10]
miR-146a	*EGFR, IRAK1, TRAF6*	Regulates inflammation and tumor progression	[11]
miR-200 family	*ZEB1, ZEB2, E-cadherin*	Suppresses EMT and inhibits metastasis	[12]
miR-335	*SOX4, TNC, TGFBR2*	Modulates migration, invasion, and EMT	[13]
miR-9	*CDH1, CDH2, MMP14*	Controls migration, invasion, and differentiation	[14]

**EMT:** Epithelial-mesenchymal transition;

**Table:** Table2. miRNAs as Prognostic Biomarkers for Prediction of Brain Metastasis in Patients with BC

**miRNAs**	**Targeted Genes**	**Functions**
miR-10b	*HOXD10, TP53*	Enhances invasion, migration, and EMT
miR-125b	*HER-2, ERBB2*	Inhibits HER-2 expression and proliferation
miR-126	*SPRED1, CRK, IRS-1*	Regulates cell adhesion, migration, and angiogenesis
miR-146a	*TRAF6, IRAK1*	Modulates inflammation and immune responses
miR-200 family	*ZEB1, ZEB2, E-cadherin*	Inhibits EMT and suppression of metastasis
miR-205	*ZEB1, ZEB2, E-cadherin*	Regulates EMT and inhibits metastasis
miR-210	*EFNA3, HOXA1, RAD52, TP53*	Promotes angiogenesis and metastasis
miR-221/222	*CDKN1B (p27), TIMP3, ICAM1, PTEN*	Facilitates proliferation, angiogenesis, and invasion
miR-375	*PDK1, SP1, JAK2*	Inhibits invasion and migration
miR-520c-3p	*EGFR, HER-2*	Targets EGFR and HER-2 to inhibit proliferation

**EMT:** Epithelial-mesenchymal transition;**HER-2:**Human epidermal growth factor receptor 2;**EGFR:**Epidermal growth factor receptor

**Table:** Table3. Pre-Clinical Studies with Targeted miRNA for Inhibition Brain Metastasis in Patients with BC [18]

**Treatment Approaches**	**Findings**
Delivery of synthetic miR-203	Inhibition of brain metastasis and reduction in tumor growth in a mouse model
Inhibition of miR-19a	Suppression of brain metastasis, reduced angiogenesis, and increased survival in mice
Delivery of miR-7	Inhibition of brain metastasis by targeting *KLF4 * and * MMP-2 * in mice
Anti-miR-10b treatment	Reduction in brain metastasis, inhibition of invasion, and increased survival in mice
Delivery of miR-33b	Suppression of brain metastasis and inhibition of migration in mouse
Inhibition of miR-203	Suppression of brain metastasis, reduced invasiveness, and increased survival in mice
Delivery of miR-20a	Inhibition of brain metastasis, suppression of EMT, and prolonged survival in mice

**KLF4:**Krüppel-like factor 4;**MMP-2:**Matrix metalloproteinase-2;**EMT:** Epithelial-mesenchymal transition;

## Conflict of Interest

None.
